# Supplementary material for: Contribution of malaria and sickle cell disease to anaemia among children aged 6–59 months in Nigeria: a cross-sectional study using data from the 2018 Demographic and Health Survey
Source: BMJ Open. 2022 Nov 15;12(11):e063369. doi: 10.1136/bmjopen-2022-063369 (PMC9670918; doi:10.1136/bmjopen-2022-063369)
Supplement: Supplementary data [file bmjopen-2022-063369supp001.pdf]

Supplemental tables

Table S1. Predictors of anaemia with RDT-positive as reference. Multivariate logistic regression model for predicting mild-to-severe (left columns), moderate-to-severe (middle columns), and severe anaemia (right columns). The effect of risk factors expressed as odds ratios.

	Adjusted haemoglobin <110 g/L (anaemia)			***Adjusted haemoglobin <100 g/L (moderate-to-severe anaemia)***			***Adjusted haemoglobin <70 g/L (severe anaemia)***		
	Odds ratio	***(2.5%, 97.5%)***	***p***	***Odds ratio***	***(2.5%, 97.5%)***	***p***	***Odds ratio***	***(2.5%, 97.5%)***	***p***
age in years	0.68	(0.65,0.71)	<0.001	0.71	(0.69,0.74)	<0.001	0.73	(0.67,0.80)	<0.001
female	0.86	(0.77,0.96)	0.009	0.84	(0.76,0.93)	<0.001	0.87	(0.67,1.14)	0.31
HBB/malaria status									
HbAA/+ (ref)									
HbAS/+	0.84	(0.64,1.08)	0.18	0.84	(0.70,1.00)	0.051	0.48	(0.31,0.72)	<0.001
HbAC/+	0.72	(0.40,1.29)	0.26	0.98	(0.60,1.59)	0.93	0.14	(0.02,1.02)	0.052
HbSC/+	*	*	*	4.53	(1.01,20.35)	0.05	4.78	(0.85,26.71)	0.08
HbSS/+	*	*	*	12.33	(2.73,55.81)	0.001	15.54	(6.24,38.74)	<0.001
HbAA/-	0.25	(0.21,0.29)	<0.001	0.20	(0.18,0.22)	<0.001	0.09	(0.06,0.14)	<0.001
HbAS/-	0.32	(0.19,0.36)	<0.001	0.26	(0.22,0.31)	<0.001	0.06	(0.03,0.15)	<0.001
HbAC/-	0.29	(0.24,0.54)	<0.001	0.28	(0.17,0.47)	<0.001	#	#	#
HbSC/-	2.97	(0.43,20.35)	0.27	0.48	(0.12,1.98)	0.31	#	#	#
HbSS/-	17.18	(2.36, 125.03)	0.005	4.53	(2.08,9.89)	<0.001	6.95	(3.32,14.55)	<0.001
rural (urban ref)	0.99	(0.85,1.14)	0.86	0.92	(0.80,1.05)	0.22	1.62	(1.13,2.31)	0.008
wealth									
richer/richest (ref)									
middle	1.03	(0.88,1.20)	0.71	1.09	(0.93,1.28)	0.28	0.96	(0.60,1.53)	0.87
poorer	1.33	(1.09,1.63)	0.005	1.34	(1.11,1.62)	0.003	1.27	(0.82,1.97)	0.29
poorest	1.71	(1.36,2.14)	<0.001	1.51	(1.24,1.84)	<0.001	1.56	(0.97,2.49)	0.06
mother's education									
none or primary (ref)									
Secondary or higher	0.85	(0.73,1.00)	0.05	0.85	(0.74,0.98)	0.03	0.95	(0.68,1.33)	0.76

* indicates odds ratios could not be estimated because all observed children in this category were anaemic

# odds ratios could not be estimated because no observed children in this category were severely anaemic.

Table S2. Predictors of mild-to-severe, moderate-to-severe, and severe anemia without using sickle cell status.

	Mild-to-severe			*Moderate-to-severe*			*Severe*		
	Odds ratio	***(2.5, 97.5%)***	***p***	***Odds ratio***	***(2.5, 97.5%)***	***p***	***Odds ratio***	***(2.5%, 97.5%)***	***p***
age in years	0.68	(0.65, 0.71)	<0.001	0.72	(0.69, 0.74)	<0.001	0.74	(0.68, 0.81)	<0.001
female	0.87	(0.78, 0.97)	0.01	0.85	(0.77, 0.94)	<0.01	0.90	(0.69, 1.17)	0.43
RDT positive	3.69	(3.23, 4.21)	<0.001	4.48	(4.02, 4.98)	<0.001	6.97	(5.00, 9.73)	<0.001
rural (ref: urban)	0.98	(0.84, 1.13)	0.75	0.91	(0.79, 1.04)	0.18	1.68	(1.18, 2.40)	0.004
wealth									
richer/richest (ref)	--			--			--		
middle	1.03	(0.88, 1.21)	0.71	1.09	(0.93, 1.28)	0.28	0.96	(0.61-1.51)	0.85
poorer	1.35	(1.11, 1.65)	<0.01	1.36	(1.12, 1.64)	<0.01	1.37	(0.89-2.10)	0.16
poorest	1.71	(1.37, 2.14)	<0.001	1.52	(1.25, 1.85)	<0.001	1.57	(0.99-2.50)	0.06
mother's education									
None or primary (ref)	--			--			--		
Secondary or higher	0.87	(0.74, 1.01)	0.07	0.86	(0.75, 1.00)	<0.05	1.02	(0.73-1.43)	0.90

Table S3. Predictors of haemoglobin concentration with RDT-positive as reference. Multivariate linear regression results to predict adjusted haemoglobin concentration. The baseline haemoglobin concentration is in the first row, with the following rows summarizing the effect of risk factors.

	g/L	***(2.5%,97.5%)***	***p***
Baseline concentration	86.36	(85.00,87.72)	<0.001
age in years	2.74	(2.50,2.97)	<0.001
Female (ref: male)	1.23	(0.61,1.85)	<0.001
HBB/malaria status			
HbAA/+ (ref)	--		
HbAS/+	2.17	(0.90,3.45)	<0.001
HbAC/+	3.51	(0.26,6.75)	0.03
HbSC/+	-14.92	(-22.74,-7.10)	<0.001
HbSS/+	-24.32	(-30.64,-18.00)	<0.001
HbAA/-	12.93	(12.10,13.76)	<0.001
HbAS/-	11.06	(10.01,12.12)	<0.001
HbAC/-	11.78	(8.98,14.58)	<0.001
HbSC/-	0.58	(-2.97,4.14)	0.74
HbSS/-	-15.98	(-20.44,-11.53)	<0.001
rural (ref: urban)	-0.03	(-0.87, 0.81)	0.94
wealth			
richer/richest (ref)	--		
middle	-0.15	(-1.05, 0.76)	0.75
poorer	-1.89	(-3.04, -0.74)	0.001
poorest	-3.80	(-5.10, -2.50)	<0.001
mother's education			
none or primary (ref)	--		
Secondary or higher	1.12	(0.28,1.97)	0.009

Supplemental figures

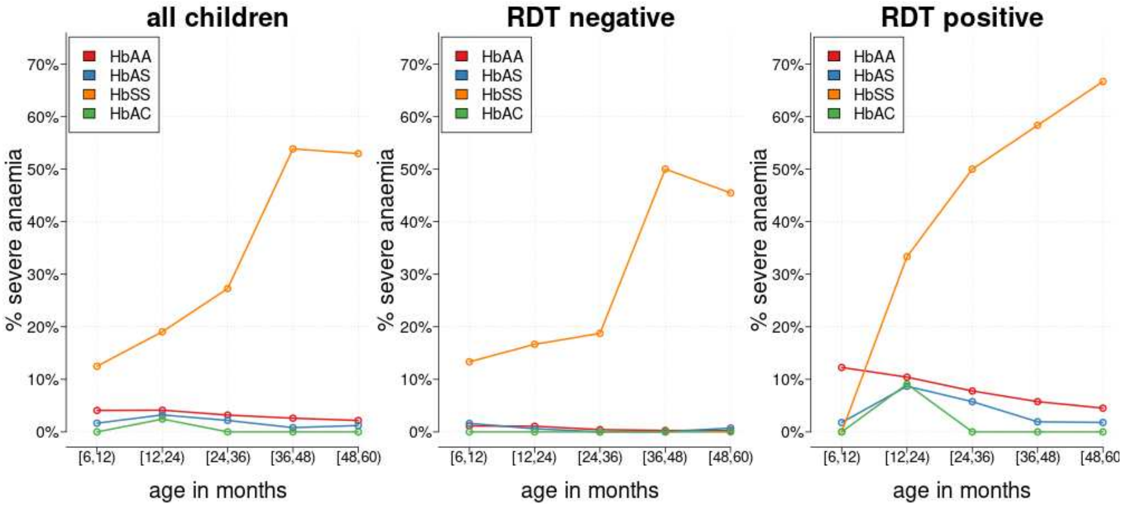

Figure S1. Severe anemia by age and sickle cell genotype. Among children with HbSS, the prevalence of severe anemia increases dramatically with age. Among children without HbSS, the prevalence of severe anemia decreases with age. Also note the difference between HbAA (red) and HbAS (blue) among RDT-positive children.

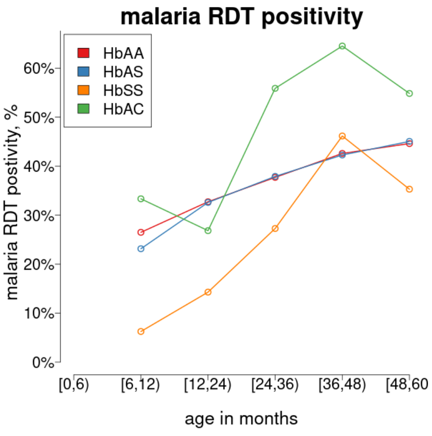

Figure S2. Malaria positivity by age and sickle cell genotype.
